# Reduced lung function is independently associated with increased risk of type 2 diabetes in Korean men

**DOI:** 10.1186/1475-2840-11-38

**Published:** 2012-04-24

**Authors:** Chang-Hee Kwon, Eun-Jung Rhee, Jae-Uk Song, Jung-Tae Kim, Hyon Joo Kwag, Ki-Chul Sung

**Affiliations:** 1 Department of Cardiology, Asan Medical Center, University of Ulsan College of Medicine, Seoul, South Korea; 2 Department of Internal Medicine, Division of Endocrinology and Metabolism, Kangbuk Samsung Hospital, Sungkyunkwan University School of Medicine, Seoul, South Korea; 3 Department of Internal Medicine, Division of Pulmonary and Critical Care Medicine, Kangbuk Samsung Hospital, Sungkyunkwan University School of Medicine, Seoul, South Korea; 4 Department of Thoracic and Cardiovascular Surgery, Kangbuk Samsung Hospital, Sungkyunkwan University School of Medicine, Seoul, South Korea; 5 Department of Radiology, Kangbuk Samsung Hospital, Sungkyunkwan University School of Medicine, Seoul, Korea; 6 Division of Cardiology, Department of Internal Medicine, Kangbuk Samsung Hospital, Sungkyunkwan University School of Medicine, 108 Pyung-dong, Jongno-Ku, Seoul, 110-746, South Korea

**Keywords:** Lung function, Type 2 diabetes mellitus, Retrospective study

## Abstract

**Background:**

Reduced lung function is associated with incident insulin resistance and diabetes. The aim of this study was to assess the relationship between lung function and incident type 2 diabetes in Korean men.

**Methods:**

This study included 9,220 men (mean age: 41.4 years) without type 2 diabetes at baseline who were followed for five years. Subjects were divided into four groups according to baseline forced vital capacity (FVC) (% predicted) and forced expiratory volume in one second (FEV_1_) (% predicted) quartiles. The incidence of type 2 diabetes at follow-up was compared according to FVC and FEV_1_ quartiles.

**Results:**

The overall incidence of type 2 diabetes was 2.2%. Reduced lung function was significantly associated with the incidence of type 2 diabetes after adjusting for age, BMI, education, smoking, exercise, alcohol, and HOMA-IR. Both FVC and FEV_1_ were negatively associated with type 2 diabetes (*P* < 0.05). In non-obese subjects with BMI < 25, the lowest quartile of FVC and FEV_1_ had a significantly higher odds ratio for type 2 diabetes compared with the highest quartile after adjusting for age and BMI (2.15 [95% CI 1.02-4.57] and 2.19 [95% CI 1.09-4.42]).

**Conclusions:**

Reduced lung function is independently associated with the incidence of type 2 diabetes in Korean men.

## Background

Obesity is a serious problem that causes various metabolic diseases [1,2]. Type 2 diabetes is a representative metabolic diseases that is caused by obesity, and recent survey presents rapidly increasing prevalence of diabetes in Koreans [3].

The association between obesity and respiratory dysfunction is as old as recorded history [4], and obstructive sleep apnea could be the link that associates obesity with reduced lung function [5]. In a very recent study by Fredheim et al. [6] showed direct associations of prediabetes and type 2 diabetes with obstructive sleep apnea in extremely obese subjects, suggesting possible link between sleep apnea, reduced lung function and glucose intolerance. From the above algorithm, reduced lung function as measured by forced vital capacity (FVC) or forced expiratory volume in one second (FEV_1_) could be suggested as the predictor for type 2 diabetes development [7-11]. These studies suggest that lung dysfunction may be associated with the development of type 2 diabetes. However, the causal direction between reduced lung function and diabetes, as well as the underlying mechanism to explain this association, remains unclear.

Although a number of Asian studies have also revealed a significant association between reduced lung function and insulin resistance, metabolic syndrome and type 2 diabetes [12-15], they were limited by their cross-sectional nature. Here we investigated whether reduced lung function was a significant predictor of incident type 2 diabetes in Korean men by a five-year follow-up study.

## Subjects and methods

### Subjects

Study subjects were selected from a total of 10,965 men who visited Kangbuk Samsung Hospital Health Promotion Center for health examinations in both 2003 and 2008. The purpose of the medical health checkup program is to promote the health of the employees through regular health checkups and to enhance early detection of existing diseases, if any. Most of the examinees are the employees and family members of various industrial companies from all around the country. The costs of the medical examinations are largely paid for by their employers, and a considerable proportion of the examinees undergo examinations annually or biannually. We took advantage of this opportunity to conduct a follow-up study.

Among the potential study subjects, 1,745 individuals were excluded; 139 individuals had a history of type 2 diabetes, 237 individuals had fasting plasma glucose ≥ 7.0 mmol/L (126 mg/dl) in 2003 (baseline visit), 31 individuals did not have available FEV_1_ or FVC level measurements, and 1,338 individuals had no available data regarding education, alcohol, smoking or exercise. Finally, 9,220 men (mean age: 41.4 years, range: 24 to 82 years) who did not have type 2 diabetes in 2003 (baseline visit) were enrolled in the study and were followed up for five years. The study subjects were divided into quartiles according to the baseline percentage of predicted values (% predicted) for FEV_1_ or FVC. Based on FEV_1_ (% predicted), the resulting four categories were as follows: ≤94.6% in quartile 1, 94.6-105.5% in quartile 2, 105.5-119.9% in quartile 3, and >119.9% in quartile 4. The subjects were similarly divided into quartiles based on FVC (% predicted): ≤89.4% in quartile 1, 89.4-98.6% in quartile 2, 98.6-109.1% in quartile 3, and ≥109.1% in quartile 4. We then compared the incidence of type 2 diabetes according to different FVC (% predicted) and FEV_1_ (% predicted) groups after a follow-up survey.

Medical and medication history, smoking status (current-, ex-, or non-smoker), alcohol consumption (g/day), regular exercise (≥ once per week), and education status were assessed using the same standard questionnaire in 2003 and 2008. Blood pressure (BP) was measured with a standard sphygmomanometer following at least five minutes of seated rest. Height and weight were estimated using automated instruments, with individuals wearing light clothing and no shoes. Body mass index (BMI) was calculated as body weight (kilograms) divided by height squared (meters^2^).

The study protocol conformed to ethical guidelines of the 1975 Declaration of Helsinki, and accordingly the Kangbuk Samsung Hospital Human Research Committee approved it. The Kangbuk Samsung Hospital Institutional Review Board also approved this study and each participant gave their written informed consent.

### Measurements

Morning blood samples were drawn from the antecubital vein after participants had fasted for at least 12 hours. Plasma glucose levels were determined using the hexokinase method (Advia 1650 AutoAnalyzer, Bayer Diagnostics, Leverkusen, Germany). Insulin concentrations were measured using immunoradiometric assays (RIABEAD II, Abbott, Tokyo, Japan), with intra- and inter-assay coefficients of variance of 1.2-1.9% and 1.4-3.3%, respectively. Insulin resistance was estimated using the homeostasis model assessment of insulin resistance index (HOMA-IR), which is defined as fasting insulin (μIU/ml) x fasting glucose (mmol/L)/22.5 [16]. Fasting total cholesterol (TC) and triglyceride (TG) levels were measured enzymatically using an automatic analyzer (Advia 1650 AutoAnalyzer, Bayer Diagnostics, Leverkusen, Germany). High density lipoprotein cholesterol (HDL-C) concentrations were measured using a selective inhibition technique. Levels of low density lipoprotein cholesterol (LDL-C) were determined through a homogeneous enzymatic calorimetric test. Type 2 diabetes was defined as fasting plasma glucose ≥ 7.0 mmol/L (126 mg/dL) based on 1997 American Diabetes Association criteria, current usage of diabetes medication, or positive response to the question, “Has a medical person ever told you that you had diabetes?” [17].

### Lung function assessment

Spirometry was performed as recommended by the American Thoracic Society [18] using V_max_22 (SensorMedics, Yorba Linda, CA, USA). Absolute values of FEV_1_ and FVC were obtained, and the percentage predicted values (% predicted) for FEV_1_ and FVC were calculated from the following equations obtained in a representative Korean population sample [19]:

(1) $$\begin{array}{l} \text{Predicted FVC}=-4.8434\;–(0.0000{8633\text{ x age}}^{2}\left[\text{years}\right])+\left(0.05292\text{ x height }\left[\text{cm}\right]\right)+\left(0.01095\text{ x weight }\left[\text{kg}\right]\right)\\ {\text{Predicted FEV}}_{1}=-3.4132\;–(0.0002484\text{x}{\text{age}}^{2}\left[\text{years}\right])+\left(0.04578\text{ x height }\left[\text{cm}\right]\right) \end{array}$$

The highest FEV_1_ and FVC values from three or more tests with acceptable curves were used for further analyses. The FVC (% predicted) and FEV_1_ (% predicted) were calculated by dividing the FVC (L) and FEV_1_ (L) by the predicted FVC and FEV_1_, respectively.

The reliability of the spirometry is proved in the previously published report [15].

### Statistical analysis

Data are expressed as the mean ± standard deviation (SD) for continuous variables and as percentages for categorical variables. Serum TG concentration was log-transformed for analysis to correct skewed distributions, but the values in the tables are expressed as untransformed data for easy interpretation. Comparisons of baseline cardiovascular risk factors according to the presence/absence of incident type 2 diabetes were made using Student’s *t*-test for continuous variables or the chi-square test for categorical variables. Comparisons of baseline variables between the lowest and highest quartiles of FEV_1_ (% predicted) or FVC (% predicted) were made by Student’s *t*-test or the chi-square test. Comparisons of type 2 diabetes development according to FEV_1_ (% predicted) or FVC (% predicted) quartiles were obtained from chi-square tests. Multivariable logistic regression analyses were conducted to assess the relationship between FEV_1_ (% predicted) or FVC (predicted) quartiles and the risk of incident type 2 diabetes: model 1 was adjusted for age and BMI; model 2 was adjusted as in model 1 plus education, smoking, exercise, alcohol, and insulin; model 3 was adjusted as in model 1 plus education, smoking, exercise, alcohol, and HOMA-IR. The outcome in this study was type 2 diabetes at five years and all subjects had been followed for approximately five years. Therefore, our data for the analysis was complete (not censored) in terms of outcome. For this reason, data was analyzed by logistic regression instead of using a Cox regression model. All statistical analyses were performed using PASW for Windows, version 18.0 (SPSS Inc., Chicago, IL, USA). All statistical tests were two-tailed, and *P*-values < 0.05 were considered statistically significant.

## Results

The overall incidence of type 2 diabetes in the study population was 2.2% (207 of 9,220 men). According to the quartiles of FVC (% predicted), the incidence of type 2 diabetes was 3.6% in the lowest quartile (quartile 1), 1.7% in the second (quartile 2), 2.3% in the third (quartile 3), and 1.3% in the highest quartile (quartile 4) (P < 0.0001). Moreover, the difference in incidence of type 2 diabetes according to the quartile of FEV_1_ (% predicted) was also significant [3.1% in quartile 1, 2.3% in quartile 2, 1.9% in quartile 3, and 1.7% in quartile 4 (P = 0.005)]. The overall incidence of type 2 diabetes in the study group was similar to that of the excluded subjects [2.2% (207/9,220) vs. 2.0% (35/1,745), respectively, P = 0.438].

### Clinical characteristics of the subjects according to diabetes development at follow-up

Baseline characteristics of the non-diabetes group and the diabetes group at follow-up are presented in Table 1. Individuals in the diabetes group were older and more likely to have higher mean BP, heart rate (HR), BMI, TC, TG, glucose, insulin, and HOMA-IR, and lower HDL-C, FEV_1_ (L), and FVC (L) values compared with those in the non-diabetes group. In addition, there were more subjects in the diabetes group who were obese, currently smoking, and who had a low-level of education (Table 1).

**Table 1:** Baseline characteristics of the non-diabetic and diabetic groups at follow-up

	**Non-diabetic group (n = 9,013)**	**Diabetic group (n = 207)**	***P* value**
Age (years)	41.3 ± 5.8	42.6 ± 5.6	0.002
Systolic BP (mmHg)	117.0 ± 12.4	122.6 ± 14.7	<0.0001
Diastolic BP (mmHg)	76.6 ± 9.3	80.3 ± 10.6	<0.0001
HR (n/min)	65.6 ± 8.8	68.5 ± 10.2	<0.0001
BMI (kg/m^2^)	24.4 ± 2.6	26.7 ± 3.3	<0.0001
TC (mmol/L)	5.42 ± 0.90	5.59 ± 0.94	0.007
TG (mmol/L)	1.73 ± 0.98	2.36 ± 1.64	<0.0001
HDL-C (mmol/L)	1.35 ± 0.26	1.28 ± 0.23	<0.0001
LDL-C (mmol/L)	3.16 ± 0.75	3.25 ± 0.73	0.072
Glucose (mmol/L)	5.20 ± 0.45	6.00 ± 0.50	<0.0001
Insulin (pmol/L)	50.58 ± 19.36	67.86 ± 31.88	<0.0001
HOMA-IR	1.69 ± 0.69	2.61 ± 1.24	<0.0001
FEV_1_ (L)	4.32 ± 0.81	4.13 ± 0.84	0.001
FVC (L)	4.88 ± 0.90	4.71 ± 0.86	0.010
FEV_1_ (%)	108.0 ± 18.7	103.5 ± 19.1	0.001
FVC (%)	100.2 ± 17.1	95.2 ± 15.3	<0.0001
Alcohol (g/day)	14.3 ± 16.1	16.6 ± 19.6	0.097
Weight class, n (%)			
BMI <25	5,353 (59.4)	64 (30.9)	<0.0001
BMI ≥25	3,659 (40.6)	143 (69.1)	
Smoking status, n (%)			
Non-smoker	2,858 (31.7)	50 (24.2)	0.029
Ex-smoker	2,418 (26.8)	54 (26.1)	
Current-smoker	3,736 (41.5)	103 (49.8)	
Education status, n (%)			
≤12 years	1,689 (18.7)	49 (23.7)	0.004
≤14 years	706 (7.8)	26 (12.6)	
≥16 years	6,618 (73.4)	132 (63.8)	
Regular exercise, n (%)			
None	2,221 (24.6)	47 (22.7)	0.44
<1 time/week	3,523 (39.1)	90 (43.5)	
≥1 time/week	3,269 (36.2)	70 (33.8)	
FVC (%) quartiles, n (%)			
Q 1 (≤89.4)	2,221 (24.6)	84 (40.6)	<0.0001
Q 2 (89.4-98.6)	2,265 (25.1)	40 (19.3)	
Q 3 (98.6-109.1)	2,253 (25.0)	52 (25.1)	
Q 4 (>109.1)	2,274 (25.2)	31 (15.0)	
FEV_1_ (%) quartiles, n (%)			
Q 1 (≤94.6)	2,233 (24.8)	72 (34.8)	0.005
Q 2 (94.6-105.5)	2,252 (25.0)	53 (25.6)	
Q 3 (105.5-119.9)	2,262 (25.1)	43 (20.8)	
Q 4 (>119.9)	2,266 (25.1)	39 (18.8)	

Values are mean ± SD for continuous variables and number (percentages) for categorical variables. P values for continuous variables were obtained from the Student’s *t* test. P values for categorical variables were obtained from Chi-square tests. TG was expressed as raw data but was applied for statistical analysis after natural logarithmic transformation.

BP, blood pressure; HR, heart rate; BMI, body mass index; TC, total cholesterol; TG, triglyceride; HDL-C, high-density lipoprotein cholesterol; LDL-C, low density lipoprotein cholesterol; HOMA-IR, homeostasis model assessment – insulin resistance; FEV1, forced expiratory volume in one second; FVC, forced vital capacity; Q, quartile.

### Baseline characteristics according to quartiles of FEV_1_ (% predicted) and FVC (% predicted)

The baseline characteristics of the lowest and highest quartiles of FEV_1_ (% predicted) and FVC (% predicted) were compared (Table 2). In the lowest quartile (quartile 1) of FEV_1_ (% predicted), TG, glucose and the percentage of current smokers were significantly higher compared to the highest quartile (quartile 4). However, age, HR, BMI, HDL-C, insulin and alcohol consumption were significantly lower in the lowest quartile compared to the highest quartile. On the other hand, subjects in quartile 1 of FVC (% predicted) were older, more likely to have higher BP, BMI, TG, glucose, insulin and HOMA-IR, and had lower HR and HDL-C compared to quartile 4.

**Table 2:** **Baseline characteristics according to quartiles of FEV**_**1**_**(% predicted) and FVC (% predicted)**

	**FEV_1_ (%)**			**FVC (%)**		
	**Quartile 1 (≤94.6)**	**Quartile 4 (>119.9)**	**P value**	**Quartile1 (≤89.4)**	**Quartile 4 (>109.1)**	***P* value**
n	2305	2305		2305	2305	
Age (years)	40.8 ± 5.7	41.9 ± 6.1	<0.0001	42.2 ± 6.5	40.9 ± 5.4	<0.0001
Systolic BP (mmHg)	117.5 ± 12.8	117.5 ± 12.6	0.989	118.0 ± 13.1	117.0 ± 12.2	0.008
Diastolic BP (mmHg)	77.2 ± 9.7	76.6 ± 9.3	0.039	77.5 ± 9.7	76.4 ± 9.1	<0.0001
HR (n/min)	65.1 ± 8.8	66.7 ± 9.2	<0.0001	65.5 ± 8.8	66.3 ± 9.2	0.002
BMI (kg/m^2^)	24.5 ± 2.8	24.7 ± 2.5	0.011	24.8 ± 2.8	24.3 ± 2.4	<0.0001
TC (mmol/L)	5.43 ± 0.93	5.48 ± 0.91	0.073	5.46 ± 0.92	5.44 ± 0.92	0.415
TG (mmol/L)	1.84 ± 1.14	1.72 ± 0.95	<0.0001	1.84 ± 1.14	1.68 ± 0.97	<0.0001
HDL-C (mmol/L)	1.32 ± 0.25	1.38 ± 0.26	<0.0001	1.33 ± 0.25	1.38 ± 0.26	<0.0001
LDL-C (mmol/L)	3.17 ± 0.76	3.19 ± 0.76	0.356	3.18 ± 0.74	3.17 ± 0.76	0.613
Glucose (mmol/L)	5.30 ± 0.46	5.13 ± 0.47	<0.0001	5.30 ± 0.47	5.12 ± 0.47	<0.0001
Insulin (pmol/L)	51.08 ± 20.63	52.40 ± 19.61	0.026	52.65 ± 21.16	50.94 ± 19.43	0.004
HOMA-IR	1.74 ± 0.77	1.73 ± 0.70	0.437	1.80 ± 0.79	1.68 ± 0.69	<0.0001
FEV_1_ (L)	3.46 ± 0.39	5.33 ± 0.59	<0.0001	3.57 ± 0.46	5.25 ± 0.67	<0.0001
FVC (L)	4.20 ± 0.96	5.77 ± 0.68	<0.0001	4.02 ± 0.41	5.88 ± 0.97	<0.0001
Alcohol (g/day)	13.4 ± 16.0	15.5 ± 16.6	<0.0001	14.3 ± 16.4	15.0 ± 16.6	0.166
Current smoker, n (%)	1053 (45.7)	906 (39.3)	<0.0001	972 (42.2)	935 (40.6)	0.393

Values are mean ± SD for continuous variables. P values for continuous variables were obtained from the Student’s *t* test. TG was expressed as raw data but was applied for statistical analysis after natural logarithmic transformation.

FEV1, forced expiratory volume in one second; FVC, forced vital capacity; BP, blood pressure; HR, heart rate; BMI, body mass index; TC, total cholesterol; TG, triglyceride; HDL-C, high-density lipoprotein cholesterol; LDL-C, low density lipoprotein cholesterol; HOMA-IR, homeostasis model assessment – insulin resistance.

### Analysis of future risk for incidence of type 2 diabetes after a five-year follow-up according to baseline lung function and obesity

**Figure 1:**
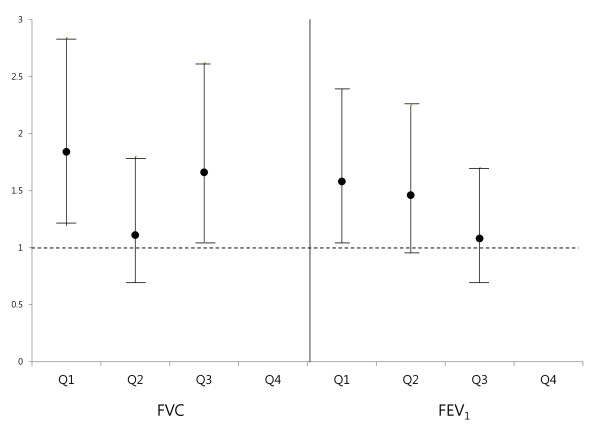
**Odds ratio for the development of type 2 diabetes according to quartiles of baseline lung function during a five-year follow-up.** FVC, forced vital capacity (% predicted); FEV1, forced expiratory volume in one second (% predicted).

In age- and BMI-adjusted logistic regression analysis (model 1), the lowest quartiles of FVC (% predicted) and FEV_1_ (% predicted) had a higher odds ratio (OR) for the incidence of type 2 diabetes compared to the highest quartile reference category (OR [95% confidence interval (CI)], 2.18[1.42-3.32] and 1.95[1.31-2.92], P < 0.0001 and P = 0.001, respectively) (Table 3, Figure 1). The OR results from models 2 and 3 were attenuated, but were still significant in FVC (% predicted) and FEV_1_ (% predicted). Moreover, analysis with model 3 including age, BMI, education, smoking, exercise, alcohol, and HOMA-IR revealed that the ORs in FVC (% predicted) and FEV_1_ (% predicted) quartile 1 were significantly higher compared with those in quartile 4 (OR [95% CI], 1.90[1.23-2.93] and 1.66[1.10-2.50], P = 0.004 and P = 0.019, respectively).

**Table 3:** Multivariable logistic regression analysis for the association between lung function and incident type 2 diabetes during a five-year follow-up

	**Type 2 diabetes**	**Model 1**		**Model 2**		**Model 3**	
	**n (%)**	**OR[95% CI]**	***P***	**OR[95% CI]**	***P***	**OR[95% CI]**	***P***
FVC							
Q 1	84 (3.6)	2.18[1.42-3.32]	<0.0001	2.00[1.30-3.07]	0.002	**1.84[1.19-2.84]**	**0.006**
Q 2	40 (1.7)	1.18[0.73-1.91]	0.487	1.16[0.72-1.87]	0.548	**1.11[0.69-1.80]**	**0.665**
Q 3	52 (2.3)	1.67[1.06-2.62]	0.026	1.64[1.04-2.59]	0.032	**1.66[1.05-2.62]**	**0.030**
Q 4	31 (1.3)	1		1		1	
*P* value	<0.0001^*^						
FEV_1_							
Q 1	72 (3.1)	1.95[1.31-2.92]	0.001	1.78[1.18-2.67]	0.006	**1.58[1.04-2.39]**	**0.030**
Q 2	53 (2.3)	1.59[1.04-2.43]	0.032	1.54[1.01-2.36]	0.047	**1.46[0.95-2.25]**	**0.082**
Q 3	43 (1.9)	1.20[0.77-1.86]	0.428	1.14[0.73-1.79]	0.555	**1.08[0.69-1.70]**	**0.734**
Q 4	39 (1.7)	1		1		1	
*P* value	0.005^*^						

^*^P value was obtained from Chi-square tests for the comparisons of the incident diabetes among quartiles of FVC (% predicted) and FEV_1_ (% predicted).

Model 1 was adjusted for age and BMI.

Model 2 was adjusted as in model 1 plus education, smoking, exercise, alcohol and insulin.

Model 3 was adjusted as in model 1 plus education, smoking, exercise, alcohol, HOMA-IR, **TC, TG, and HDL-C.**

FEV1, forced expiratory volume in one second; FVC, forced vital capacity; OR, odds ratio; CI, confidence interval; BMI, body mass index; HOMA-IR, homeostasis model assessment – insulin resistance; TC, total cholesterol; TG, triglyceride; HDL-C, high-density lipoprotein cholesterol.

We also conducted a sub-analysis of 5,417 non-obese subjects with BMI <25 (mean BMI: 22.7 ± 1.6, range: 16.1 to 24.9) to evaluate the association between lung function and type 2 diabetes in the non-obese population. In the non-obese subjects with BMI <25, quartile 1 of FVC (% predicted) and FEV_1_ (% predicted) had a significantly increased OR for type 2 diabetes compared to quartile 4 after adjusting for age and BMI (OR[95% CI], 2.15[1.02-4.57] and 2.19[1.09-4.42], P = 0.045 and P = 0.028, respectively) (Table 4).

**Table 4:** Multivariable logistic regression analysis for the association between lung function and incident type 2 diabetes in non-obese subjects (BMI < 25) during a five-year follow-up

	**Type 2 diabetes**	**Model 1**		**Model 2**		**Model 3**	
	**n (%)**	**OR[95% CI]**	***P***	**OR[95% CI]**	***P***	**OR[95% CI]**	***P***
**FVC**							
Q 1	23 (1.7)	2.15[1.02-4.57]	0.045	2.06[0.97-4.38]	0.061	**1.93[0.90-4.12]**	**0.091**
Q 2	16 (1.2)	1.60[0.72-3.54]	0.247	1.55[0.70-3.45]	0.279	**1.47[0.66-3.28]**	**0.346**
Q 3	15 (1.1)	1.50[0.67-3.36]	0.322	1.53[0.68-3.42]	0.305	**1.51[0.67-3.41]**	**0.317**
Q 4	10 (0.7)	1		1		1	
*P* value	0.142^*^						
FEV_1_							
Q 1	24 (1.8)	2.19[1.09-4.42]	0.028	2.06[1.02-4.17]	0.044	**1.87[0.92-3.80]**	**0.084**
Q 2	14 (1.0)	1.30[0.60-2.83]	0.508	1.25[0.57-2.72]	0.581	**1.17[0.53-2.57]**	**0.694**
Q 3	14 (1.0)	1.23[0.56-2.66]	0.608	1.23[0.56-2.68]	0.602	**1.19[0.54-2.60]**	**0.665**
Q 4	12 (0.9)	1		1		**1**	
*P* value	0.135^*^						

^*^P value was obtained from Chi-square tests for the comparison of incident diabetes among quartiles of FVC (% predicted) and FEV_1_ (% predicted).

Model 1 was adjusted for age and BMI.

Model 2 was adjusted as in model 1 plus education, smoking, exercise, alcohol and insulin.

Model 3 was adjusted as in model 1 plus education, smoking, exercise, alcohol, HOMA-IR, **TC, TG, and HDL-C.**

FEV1, forced expiratory volume in one second; FVC, forced vital capacity; OR, odds ratio; CI, confidence interval; BMI, body mass index; HOMA-IR, homeostasis model assessment – insulin resistance; TC, total cholesterol; TG, triglyceride; HDL-C, high-density lipoprotein cholesterol.

## Discussion

The major findings of this study are 1) the subjects with reduced lung function as measured with FVC (% predicted) and FEV_1_ (% predicted) had higher incidence of type 2 diabetes, independent of other confounding factors including age, BMI, education, smoking, exercise, alcohol and HOMA-IR; and 2) in non-obese subjects with BMI <25, decreased FVC (% predicted) and FEV_1_ (% predicted) were also inversely and significantly correlated with the incidence of type 2 diabetes. Our findings are generally consistent with previous studies that reported a significant association between reduced lung function and type 2 diabetes [8-10,20,21].

Previous studies have reported that the risk of being relatively insulin resistant, as measured by HOMA-IR, significantly increased as lung function decreased [7,9,21]. In the Normative Aging Study, 1,050 non-diabetic male subjects were followed over 20 years. Lower FVC, FEV_1_, and maximal mid-expiratory flow rate at baseline were significantly associated with risk of hyperinsulinemia and estimated insulin resistance [7]. Some epidemiologic and clinical studies have found that decreased lung function is associated with type 2 diabetes, independent of obesity [8-10,21-23]. In Asia, a number of studies have reported relationships between lung function and type 2 diabetes, insulin resistance and metabolic syndrome (MS) [12-15,24,25]. Although studies were limited by their cross-sectional design or small cohorts, they suggested that FVC and FEV_1_ could be predictive markers of incident type 2 diabetes or MS in Asian populations. Recently, The Strong Heart Study reported that reduced lung function is independently associated with diabetes, and impaired lung function presents before the development of diabetes in American Indians [23].

Type 2 diabetes is associated with insulin resistance and glucose tolerance, which could originate from visceral obesity [26]. Yeh et al. found that abdominal obesity is a significant factor affecting type 2 diabetes, MS, and pulmonary function test results [23]. Another study by Jung et al. [27] reported that nonalcoholic fatty liver disease, a well-known marker for insulin resistance and MS, was associated with reduced pulmonary function. In our study, subjects in the diabetes group had a significantly higher BMI and lower FVC and FEV_1_ (L) at baseline compared to those in the non-diabetes group. In addition, ventilatory function is partially determined by respiratory muscle strength, which may be compromised in obese subjects [28].

The association between diminished lung function and type 2 diabetes may be explained according to a common inflammatory process [29]. Obesity and metabolic syndrome are associated with elevated systemic inflammatory markers and adipocytokines [30]. Alternatively, the induction of increased oxidative activity, intracellular NF-kB and inflammatory mediators could also result in chronic hyperglycemia and an increase in collagen molecule synthesis and cross-linking via the acceleration of advanced glycation end-products, ultimately negatively influencing lung function [31]. Moreover, previous studies have indicated that proinflammatory cytokines such as C-reactive protein (CRP), fibrinogen and IL-6 may play a role in the pathogenesis of type 2 diabetes [10,13,15,23], although a recent study showed no association of glucose control with hs-CRP [32]. However, we could not address the association between inflammation and type 2 diabetes because most study subjects did not have available measurements of inflammatory markers such as CRP and IL-6.

### Limitations

We note that our study had some limitations. First, there is the possibility of selection bias because most participants were residents of an urban community, all subjects were of Korean descent, from one university hospital, and there were no women in this study. Therefore, the results of this study cannot be generalized to the worldwide population. Second, incident type 2 diabetes was defined based only on fasting glucose level or a self-reported medical history of type 2 diabetes, and no oral glucose tolerance tests were performed. Thus, the incidence of type 2 diabetes may have been underestimated in this study. Third, risk of diabetes may have been influenced by early life style factors which was not investigated in this study. Therefore, future studies including birth cohort analysis are needed to clarify the cause-effect link between reduced lung function and risk of developing diabetes. Finally, our data consisted of only baseline and five-year follow-up time points. The outcome was considered the presence of type 2 diabetes at five years. Thus, logistic regression was used for analysis instead of a Cox regression model. However, our study had the advantage of including a relatively large total number of subjects over a relatively long follow-up period.

## Conclusions

In conclusion, decreased FVC (% predicted) and FEV_1_ (% predicted) were significantly associated with the incidence of type 2 diabetes in Korean men. This result suggests that reduced lung function as measured by FVC and FEV_1_ might precede the development of type 2 diabetes. Further prospective studies will be required to confirm the association between reduced lung function and diabetes in Asian populations.

## Competing interests

The authors declare that they have no competing interests.

## Authors’ contributions

CK and ER analyzed the data, drafted the manuscript and revised the manuscript. JS and JK commented on the revision of the manuscript. KS designed the study, collected and analyzed the data. All authors have given their final approval for publication of this version of the manuscript.
